# Isolation, Selection, and Identification of Keratinolytic Bacteria for Green Management of Keratin Waste

**DOI:** 10.3390/molecules29143380

**Published:** 2024-07-18

**Authors:** Wiktoria Gerlicz, Marcin Sypka, Iga Jodłowska, Aneta M. Białkowska

**Affiliations:** Institute of Molecular and Industrial Biotechnology, Faculty of Biotechnology and Food Sciences, Lodz University of Technology, 90-537 Lodz, Poland; 243669@edu.p.lodz.pl (W.G.); marcin.sypka@dokt.p.lodz.pl (M.S.); iga.jodlowska@p.lodz.pl (I.J.)

**Keywords:** isolation, keratinases, keratin biodegradation, keratinolytic bacteria, molecular identification

## Abstract

The volume of difficult-to-process keratin waste is increasing as a result of rising global meat production. If not properly managed, this waste can contribute to environmental pollution and pose a threat to human and animal welfare. An interesting and more sustainable alternative is therefore the bioconversion of keratin using microorganisms and their enzymes. This work aimed to isolate bacteria from soil samples and zoonotic keratins and to evaluate their enzymatic capacity to degrade α- and β-keratin wastes. A total of 113 bacterial strains were isolated from environmental samples and subjected to taxonomic identification using the MALDI-TOF MS technique and to a two-step screening for proteolytic and keratinolytic activity. The ability to degrade a β-rich keratin substrate was observed in almost all of the strains isolated from soil and horsehairs. In contrast, when an α-rich keratin substrate was used, the highest levels of hydrolysis were observed only for Ker39, Ker66, Ker85, Ker100, and Ker101. Strains with the highest biodegradation potential were identified using molecular biology methods. Phylogenetic analysis of 16S rDNA gene sequences allowed the assignment of selected keratinolytic microorganisms to the genera *Exiguobacterium*, *Priestia*, *Curtobacterium*, *Stenotrophomonas*, *Bacillus*, *Kocuria,* or *Pseudomonas*. The results of this study are a promising precursor for the development of new, more sustainable methods of managing keratin waste to produce high-value hydrolysates.

## 1. Introduction

Human civilization, with its numerous activities, results in the accumulation of a vast amount of solid waste in the environment. One of the fastest-growing types of waste is keratinous materials, estimated to be several million tonnes per year [1]. Keratins are primarily derived from animal body parts and serve as waste byproducts of industrial processes, mainly from slaughterhouses, poultry farms, and leather industries [2]. If not managed properly, keratin waste can significantly impact ecosystems, contributing to environmental pollution, and pose serious hazards to human and livestock health [3].

Keratins constitute a heterogeneous family of proteins and are the third most abundant biomass in nature, after chitin and cellulose. This polymer forms solid structures, held together by disulfide bonds formed between the thiol groups (–SH) of cysteine amino acid residues, as well as hydrogen bonds and hydrophobic interactions [4]. They are characterized based on their secondary structures (mainly α-helices and β-sheets), sulfur content (soft and hard), amino acid composition (basic, acidic, or neutral), molecular weight, and source of origin [5]. The multi-level structure and high number of cross-linkages between various types of keratins result in a high resistance to mechanical, chemical, and physical factors [6]. These hard-to-degrade proteins are mostly disposed of through landfilling or incineration, which is ecologically disadvantageous due to the apparent energy loss and the production of large amounts of carbon dioxide [7]. The most promising alternatives to these techniques are biotechnological methods using keratinolytic microorganisms or more controlled hydrolysis with cell-free keratinase extracts and purified keratinases [8]. The complex and recalcitrant structure of keratin requires synergistic interactions of different types of keratinolytic enzymes to be effectively decomposed.

Keratinases (E.C. 3.4.21) are extracellular enzymes capable of degrading keratin [9]. They are produced by microorganisms, including bacteria, and filamentous fungi, including dermatophytic species. The main industrial producers of keratinases are strains belonging to the genus *Bacillus* (e.g., *B. cereus*, *B. subtilis*, *B. pumilus*, *B. stearothermophilus*, *B. licheniformis*, *B. coagulans*). Other promising producers include Gram-positive bacteria such as *Lysobacter*, *Nesternokia*, *Kocuria*, *Microbacterium*, and *Streptomyces* and some Gram-negative bacteria, e.g., *Xanthomonas*, *Aeromonas*, *Stenotrophomonas*, *Serratia*, *Chryseobacterium*, and *Vibrio* [8,10,11,12]. Keratinolytic proteases have also been isolated from extremophilic microorganisms, including representatives such as *Pseudoalteromonas*, *Colwellia*, *Flavobacterium*, *Shewanella*, *Fervidobacterium*, *Thermoanaerobacter*, and *Nesternokia* [8,9].

The industry has a keen interest in microorganisms capable of producing large amounts of efficient extracellular keratinases. However, the utility of these enzymes is currently underexploited due to the limited availability of keratinolytic protease producers. Isolating novel strains that produce significant yields of well-performing enzymes with versatile substrate specificity, increased stability at elevated temperatures and/or pH values, and higher tolerance to feedback inhibition is essential for the development of a viable industrial fermentation [9,13].

In this study, keratinolytic bacteria were isolated from soil and horsehair samples. This report describes the identification of these bacteria based on MALDI-TOF MS and the molecular 16S rDNA method. The studies also determined the abilities of microorganisms to hydrolyze casein and keratin waste substrates, such as chicken feathers (CHFs), horsehair (HH), and dog hair (DH). The highest keratinolytic activity was observed in *Exiguobacterium*, *Bacillus* and *Kocuria* genera. Strains isolated within this research, thanks to their enzymatic abilities, can be regarded as innovative and environmentally friendly tools with potential application in the rational management of keratin waste and associated technologies. Such strategies are in line with the principles of green chemistry and a circular economy.

## 2. Results

### 2.1. Isolation and Preliminary Identification of Microorganisms

Bacterial colonies were isolated from environmental samples (Table 1) during cultivation on an LB medium in various temperatures (10 °C, 20 °C, and 30 °C).

**Figure 1:**
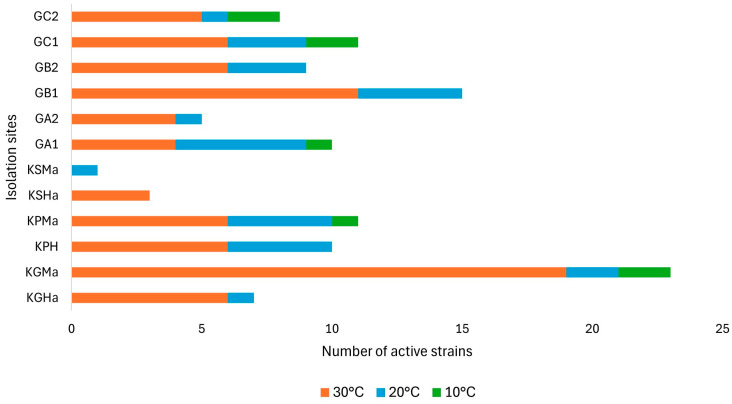
Temperature- and isolation-site-dependent number of isolated strains. Horsehair (KGHa, KGMa, KPH, KPMa, KSHa, KSMa) and soil samples (GA1, GA2, GB1, GB2, GC1, GC2).

The total number represented 113 bacterial strains: 55 isolated from horsehair samples and 58 from soil. In the presented research, the highest numbers of bacterial strains were obtained from winter coat sample KGMa (23 strains), greenhouse soil samples GB1 and GC1 (15 and 11 strains, respectively), and fetlock horsehair sample KPMa (also 11 strains). While soil samples are commonly used to isolate new microbial strains, horsehair microbiome studies tend to focus on identifying pathogens in sick animals for veterinary purposes, because the isolates are tested almost exclusively for antibiotic resistance [14]. Studies focusing on other properties of microorganisms isolated from horses are quite rare. The highest number of strains was isolated at 30 °C and consisted of 76 isolates. Significantly lower numbers of isolates were obtained at 20 °C and 10 °C, which were 29 and 8 isolates, respectively (Figure 1). The further testing of strains was carried out at the temperatures of their isolation.

**Figure 2:**
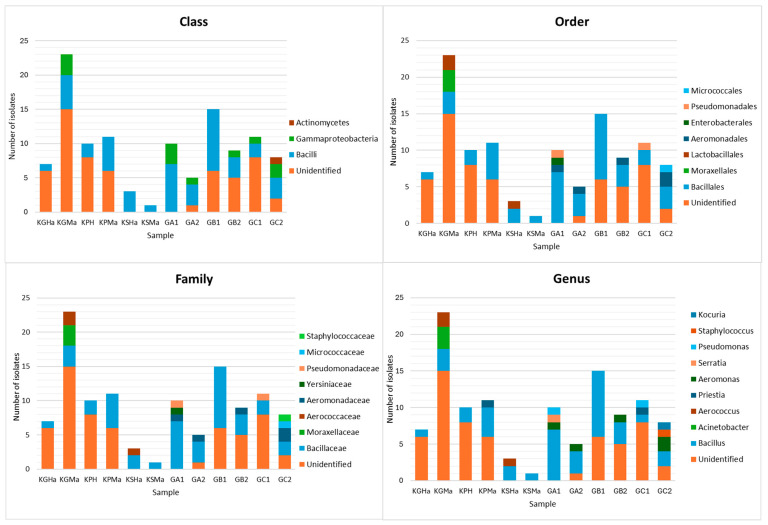
The biodiversity of strains isolated from horsehair (KGHa, KGMa, KPH, KPMa, KSHa, KSMa) and soil samples (GA1, GA2, GB1, GB2, GC1, GC2). The isolates were identified using matrix-assisted laser desorption/ionization time-of-flight mass spectrometry (MALDI-TOF MS). Only identification scores ≥ 70% were regarded as sufficient and used for biodiversity plots.

All isolated strains were subjected to preliminary taxonomic identification using matrix-assisted laser desorption/ionization time-of-flight mass spectrometry (MALDI-TOF MS). The MALDI-TOF protein mass fingerprinting was implemented in this research as a relatively fast and cost-effective technique, gaining more recognition in the field of microbial identification [15,16,17]. The results obtained in this step are available in Supplementary Table S1. Only strains with MALDI identification (ID) score ≥ 70% were regarded as sufficient and used for the generation of biodiversity plots (Figure 2).

Identified bacteria were representatives of classes Bacilli (44 strains), Gammaproteobacteria (11 strains), and Actinomycetes (1 strain). The majority (86%) of Bacilli class isolates belonged to the genus *Bacillus*—common constituent of animal, plant, water, and soil microbiomes, similarly to the identified *Aeromonas* (5 strains) [18], *Kocuria* (1 strain) [19], *Serratia* (1 strain) [20], *Pseudomonas* (2 strains) [21], and *Priestia* (2 strains) isolates [22]. Moreover, many isolated strains, belonging to the *Pseudomonas*, *Staphylococcus* and *Acinetobacter* genera, have been previously recognized as part of a healthy and wound-related horse microbiome [23,24,25]. Bacteria from *Bacillus*, *Priestia*, *Kocuria* and *Pseudomonas* genera have already been associated with positive ecological roles, including the development of soil fertility [26,27], nitrogen fixation [28], the bioremediation of hydrocarbon compounds and heavy metals [19,29], and animal and plant health and growth promotion [28,30,31,32]. However, as with many representatives of *Aeromonas*, *Staphylococcus,* and *Serratia* [33], some of the *Bacillus* and *Pseudomonas* species are also known as pathogenic and opportunistic microorganisms [34,35]. Of all isolated bacterial strains, almost 51% (57 strains) were not identified with the chosen preliminary method. This can be the result of the high diversity of environmental isolates and significant differences in their proteomic makeup in comparison to reference (often collection or clinical) strains, typically used for the development of MALDI-TOF databases [36,37]. To increase the efficiency and reliability of MALDI-TOF MS identification, different methods or more specific, custom-made databases could be used or developed [16,37,38,39].

### 2.2. Evaluation of Proteolytic Activity of Isolated Strains

**Figure 3:**
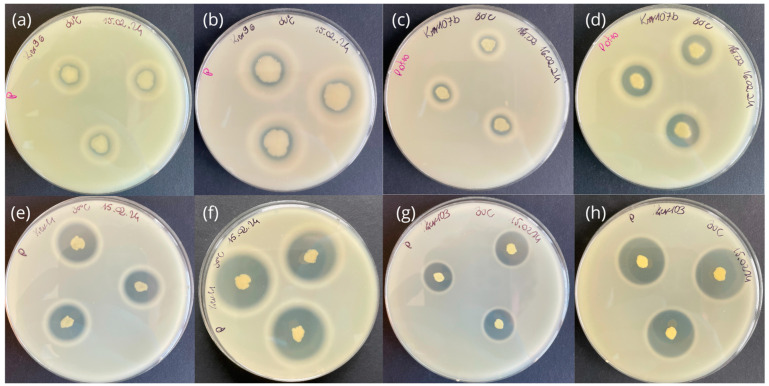
Selected strains exhibiting proteolytic activity on LB agar medium supplemented with skimmed milk after 24 h and 48 h of incubation at 30 °C. Ker96 showed low proteolytic activity after (**a**) 24 h (EAI = 0.19) and (**b**) 48 h (EAI = 0.26). Ker107b exhibited medium proteolytic activity after (**c**) 24 h (EAI = 0.65) and (**d**) 48 h (EAI = 1.10). Ker11 revealed high proteolytic activity after (**e**) 24 h (EAI = 1.94) and (**f**) 48 h (EAI = 2.50). Strain Ker103 also demonstrated high proteolytic activity within (**g**) 24 h (EAI = 1.87) and (**h**) 48 h (EAI = 2.94).

In the search for strains exhibiting keratinolytic activity, cultivation on a medium enriched with skimmed milk is a well-established method of primary selection [40,41,42,43]. Most of the identified keratinase producers also show the ability to hydrolyze simple protein substrates, including casein [44,45,46]. This strategy was also implemented in the present study. A screening test on agar plates containing skimmed milk was conducted, and the enzymatic activity index (EAI) values were calculated. A total of 106 out of 113 isolates were observed to exhibit proteolytic activity after 48 h of cultivation (Figure 3). Only for seven strains were clear zones around colonies not observed, including six horsehair isolates and one soil isolate.

A predominant majority (~98%) of the soil-derived isolates showed some degree of proteolytic activity. The ability to produce soil proteases is characteristic not only for pathogenic microorganisms, but also for typical destruents living in such environments [47]. Proteases of microbial origin are the most abundant among proteolytic enzymes in the soil [48]. Due to their unique kinetic properties and responses to environmental factors in different ecosystems, proteases are considered important in protein mineralization processes, resulting in an improvement in soil properties [47,49]. In many ecosystems, proteolysis plays a crucial role in nitrogen cycling by converting nitrogen from large biopolymers into a simpler form, which is more accessible for microbial and plant uptake [49]. Microbial proteases are also involved in carbon cycling, which is likely to be their primary function. Around 85–90% of all organic carbon decomposition is attributed to microorganisms living in the soil, making them an inherent part of most ecosystems [50]. Furthermore, protease production by microbes is essential for multispecies interactions, including symbiotic and antagonistic behaviors [51,52,53].

**Figure 4:**
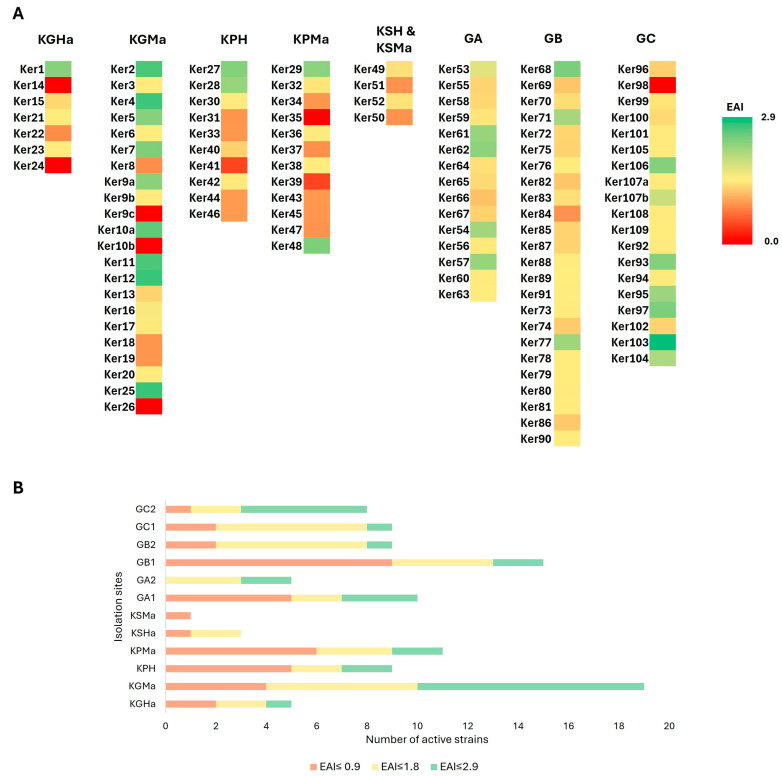
Proteolytic activity of isolated bacterial strains (**A**) with total number of active strains (EAI > 0) (**B**). The proteolytic activity was measured by the translucence around grown bacterial colonies on agar plates containing skimmed milk and expressed as enzymatic activity index (EAI) after 48 h of incubation.

Among the isolated strains, approximately 94% exhibited the ability to synthesize peptidases of varying activity (Figure 4). The highest proteolytic activity, exceeding EAI = 1.8, was observed for ten isolates: seven corresponded to horse-related samples (Ker2, Ker4, Ker7, Ker10a, Ker12, Ker25, Ker48) and three to soil samples (Ker68, Ker97, Ker103). Seven strains, Ker9c, Ker10b, Ker14, Ker24, Ker26, Ker35, and Ker98, were regarded as non-proteolytic (EAI = 0 after 48 h at given temperature; Supplementary Table S1). However, they might exhibit such activity after a longer incubation time or when cultured with proteinaceous substrates other than casein (present in skimmed milk), e.g., albumin, gelatine, fibrin, elastin, and collagen [54,55]. This might be connected to various substrate specificities of known keratinolytic enzymes [56]. Therefore, in the next stage of this research, all isolated strains were tested for keratinolytic activity by submerged cultivation with keratin waste substrates.

### 2.3. Evaluation of Keratinolytic Aptitude in the Degradation of Waste Substrates

**Figure 5:**
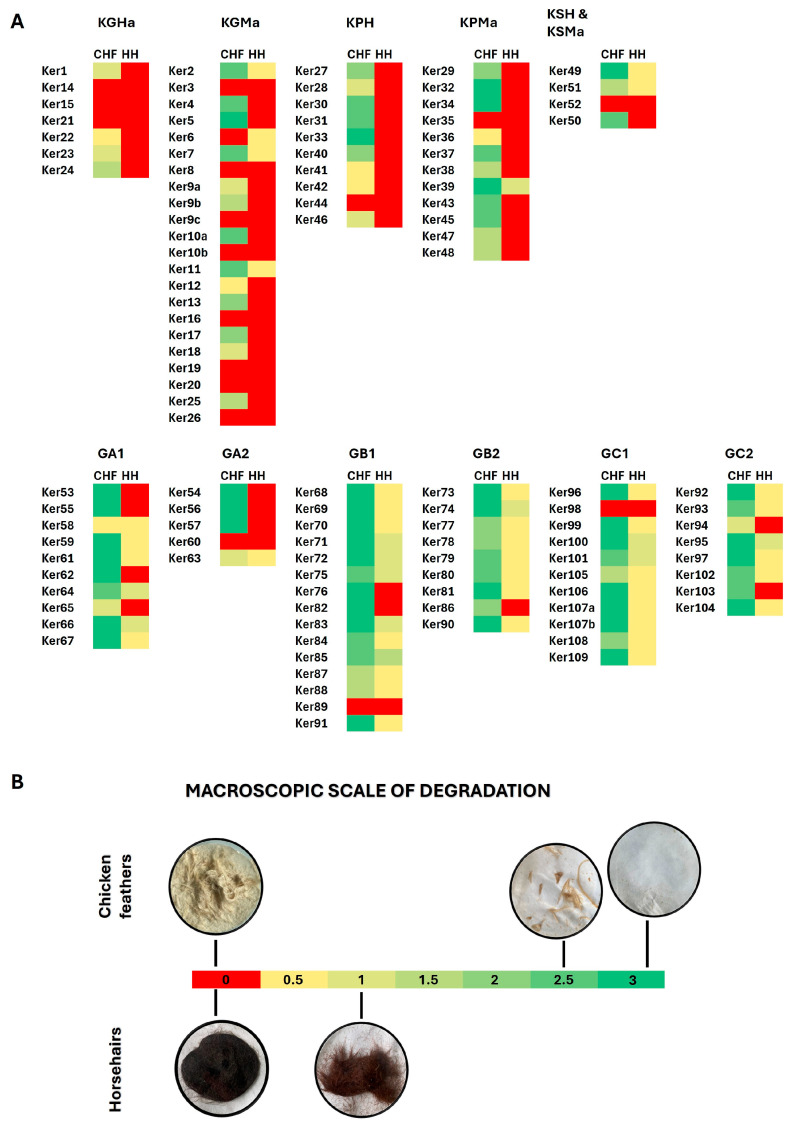
Keratinolytic activity of isolated strains on two different substrates (chicken feathers and horsehair) (**A**). The ability to degrade α- and β-rich keratin substrate was measured macroscopically, scaled from 0 to 3 (**B**).

The second stage of screening involved the cultivation of isolated strains on media containing keratin substrates, such as chicken feathers (CHFs), horsehair (HH), and dog hair (DH). After seven days of incubation (140 rpm at 10 °C, 20 °C, or 30 °C), 16 of the 113 isolates were regarded as incapable of hydrolyzing any of the used substrates (85.8% active strains) (Figure 5). In the case of DH decomposition, a semi-positive result was obtained for four isolates derived from soil (Ker75, Ker76, Ker79, Ker81), for which we only observed a higher turbidity of the culture medium. The remaining strains showed no ability to hydrolyze this substrate. Significantly, 84% of the tested strains were able to degrade the β–keratin substrate (CHFs). Most of those strains were soil bacteria. With HH as the carbon source, the only macroscopic changes that were observed were hair fragmentation, as well as a change in the color and turbidity of post-culture liquid. Complete degradation of this α-keratin-rich substrate was not observed for any strain.

There is a noticeable difference between isolates derived from soil and horsehair samples. The latter are mostly unable to degrade horsehair. This can correlate significantly with their origin, as healthy animal microbiota should not exhibit substantial keratinolytic abilities towards host organisms and can be related to inhibitory effects of the metabolism of the host organism and its cutaneous microbiota on the growth and spread of potentially pathogenic microorganisms utilizing proteases and keratinases as virulence factors. The only exception was strain Ker39, which was revealed to possess a higher ability to degrade horsehair than other horse-derived isolates. In contrast, most of the soil isolates were able to degrade this α-keratin-rich HH substrate to various extents. Moreover, Ker66, Ker71, Ker85, and Ker101 strains were able to catalyze the hydrolysis of both α- and β-rich keratin substrates (HH and CHFs, respectively). Of all 113, only 15 isolates did not exhibit a keratinolytic potential in response to any substrate under tested conditions, six of which did not possess observable proteolytic activity during cultivation on agar test plates with skimmed milk (see Section 2.2). Furthermore, two isolates Ker39 and Ker41 with low proteolytic activity (not exceeding EAI = 0.09) exhibited keratinolytic activity towards at least one keratin waste substrate. Strain Ker39 had high and medium activities towards CHFs and HH, respectively, and strain Ker41 had a medium activity towards CHFs. Interestingly, even though clear zones around colonies of isolate Ker24 were not observed when cultivated on skimmed milk agar, the strain showed the ability to hydrolyze CHFs. This indicates that the skimmed milk agar plate test, though commonly used, might not always allow for the elimination of non-keratinolytic strains in the preliminary screening stages.

This may be related to non-optimal test conditions for the sought-after enzymatic activity or with different molecular mechanisms of keratin degradation, based on the presence and cooperation of enzymes from various families and classes [44,57,58] with their substrate specificity determining the efficiency of degradation of waste keratins. In future studies, the effect of temperature, pH, time of incubation, specificity towards other keratin-rich substrates, or the role of substrate form (e.g., the size of the pieces used) could be further examined. Although the biochemical activity can vary greatly between keratinases, typically temperatures between 40 and 60 °C and neutral or alkaline pH (7–10) are considered optimal [59]. The changes in medium composition during the biodegradation process could also influence its kinetics. A study of several *Aphanoascus keratinophilus* strains suggests the presence of a weak correlation between the accumulation of the liberated ammonia in the medium and the inhibition of fungal keratinases after 3 weeks of culturing [60]. The above indicates that monitoring of the carbon-to-nitrogen ratio throughout the keratin degradation process might improve its effectiveness. 

Based on the results from screening on proteolytic and keratinolytic activity followed by MALDI-TOF, 29 strains, 8 of which were from horsehair samples and 21 were from soil samples, were selected for molecular taxonomic identification as potential producers of keratinolytic enzymes with 16S rDNA sequencing.

### 2.4. Molecular Identification and Phylogenetic Analysis of Selected Strains

Molecular identification based on sequencing of the 16S rDNA fragment was performed to accurately confirm the taxonomic affiliation of the selected 29 bacterial strains (Supplementary Table S2). The horse isolates with keratolytic potential belonged to genera *Exiguobacterium*, *Mammalicoccus* (formerly *Staphylococcus*), *Curtobacterium*, and *Priestia*. Among the soil isolates, 12 corresponded to the genus *Bacillus* and the remaining were classified as *Pseudomonas*, *Areomonas*, *Kocuria,* and *Stenotrophomonas*. Molecular identification confirmed the results obtained with MALDI-TOF MS. Even though 21 isolates were not identified by mass spectrometry (MALDI ID score lower than 70%), some strains, such as *Stenotrophomonas* Ker107b, *Pseudomonas* Ker87, and *Bacillus* Ker85, were classified correctly on the genus level and later confirmed by 16S rDNA sequencing. Moreover, none of the strains were misidentified. The microbial collection acquired during this study will enrich the in-house database of mass spectra of the keratinolytic strain, enabling the more accurate and efficient identification of related strains in the future. While marker gene sequencing remains the golden standard of taxonomic classification, both methods cannot be regarded as highly reliable for species-level identification without additional biochemical or bioinformatic analyses, as many close-related bacteria generate almost identical MALDI-TOF spectra and possess highly similar 16S rDNA sequences, especially in the *Bacillus* genus [39,61].

**Figure 6:**
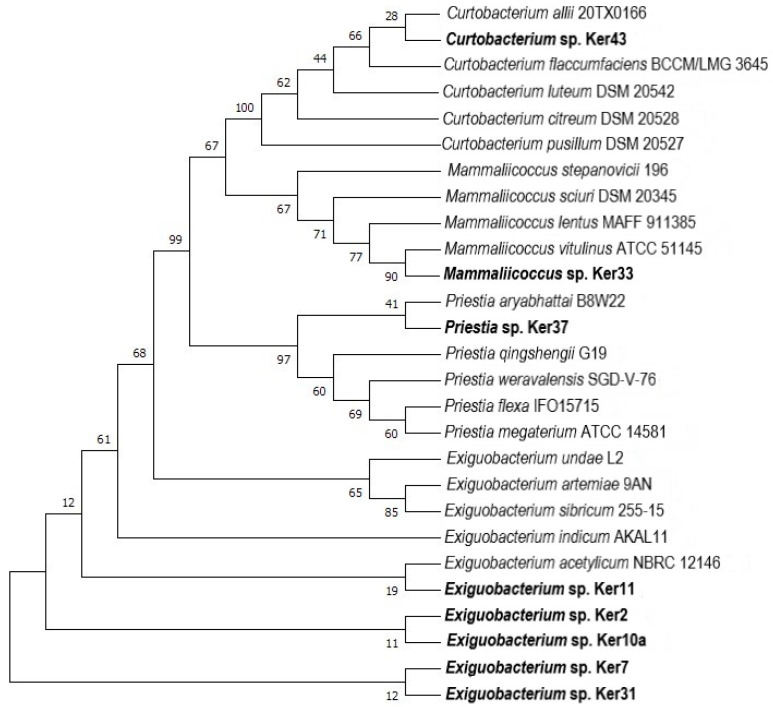
Phylogenetic analysis of strains isolated from horsehair. Phylogenetic analyses were performed in the MEGA-X program using the maximum likelihood (ML) method. Bootstraps analyses were performed using ultrafast bootstrap approximation with 1000 replicates.

**Figure 7:**
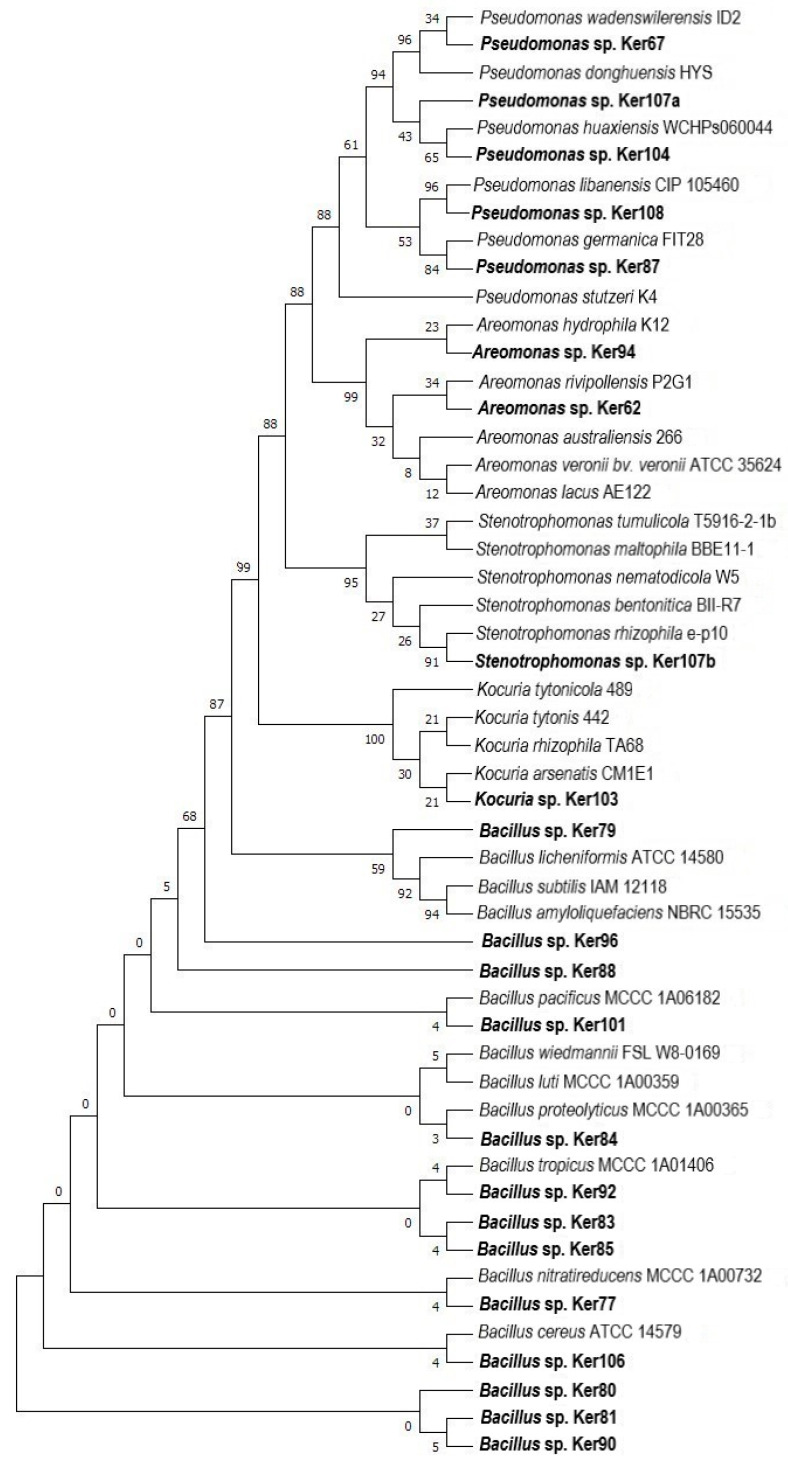
Phylogenetic analysis of strains isolated from soil. Phylogenetic analyses were performed in the MEGA-X program, using the maximum likelihood (ML) method. Bootstraps analyses were performed using ultrafast bootstrap approximation with 1000 replicates.

Phylogenetic analyses showed that all newly identified strains are phylogenetically related to the other known species of the respective genus (Figure 6 and Figure 7). The *Curtobacterium* sp. Ker43 isolated in this study, which is closely related to *Curtobacterium allii*, was the first strain with keratinolytic potential from this genus identified to date. This is in contrast to strains *Mammaliicoccus* sp. Ker33, *Priestia* sp. Ker37, and all new isolates that belong to genus *Exiguobacterium*, for which numerous literature reports indicate their keratinolytic activity. It was shown that strains of the genus *Exiguobcterium* isolated from soil samples, such as *Exiguobacterium* sp. DG1 [62] and *Exiguobacterium indicum* AKAL11 [63], were able to degrade chicken feather. None of the isolated strains belonging to this genus were closely related to keratinolytic strains described in earlier research. 

Phylogenetic analysis of the strains of the genus *Bacillus* has shown that they are mostly members of the *Bacillus cereus* group (Figure 7). Only one strain, Ker79, is closely related to *B. licheniformis*, *B. subtilis,* and *B. amyloliquefaciens*, which are some of the best known and studied keratinolytic bacteria. The distinctiveness of the *Bacillus cereus* group from *Bacillus* cluster was previously demonstrated [61]. Bacteria of the genus *Bacillus* constitute a substantial group among all keratinase-producing bacteria with many strains for which high keratinolytic activity has been proven [64,65,66,67,68]. 

Keratinolytic activity has been previously described in strains of the genus *Kocuria* [69,70], with one representative being *Kocuria rosea* LPB-3 [71] isolated from the soil of poultry processing plants or *Kocuria rizhophila* p3-3 [8]. The phylogenetic analysis of the Ker103 strain isolated in this study showed that it is closely related to the *Kocuria arsenatis*. An actinobacterial strain isolated from the *Prosopis laegivata* roots, growing on mine tailings, was resistant to a high concentration of arsenic (20 mM) [72]. However, studies showed that the isolates with keratinolytic activity also included Gram-negative bacteria. These belong to the following genera: *Pseudomonas*, *Aeromonas,* and *Stenotrophomonas*. Among Gram-negative keratinolytic isolates, 5 corresponded to the genus *Pseudomonas*, but only 2 to *Aeromonas* and 1 to *Stenotrophomonas*. The ability to hydrolyze keratin substrates has been observed in many *Pseudomonas* bacteria, among which *P. stutzeri* K4, isolated from detergent contaminated pond, is notable for its ability to degrade feathers within 5 days. Interestingly, the strain’s crude keratinase preparation was also used for the complete dehairing of goat skin in less than 24 h [73]. Moreover, phylogenetic analysis revealed a relatedness between Ker62 and *Aeromonas hydrophila* K12, a proteolytic and keratinolytic strain isolated from Brazilian soils of Atlantic Forest and exhibiting high enzyme secretion in minimal media supplemented with feather meal at various temperatures (from 30 °C to 55 °C) [7,10,74]. All the newly isolated strains represent different and equally interesting sources of potential keratinolytic enzymes that could form the basis for the development of biosynthesis of bioactive compounds from waste products, thus linking green chemistry and sustainable waste management together.

As the price of whole-genome sequencing has decreased significantly over the past few years, the analysis and comparison of good-quality bacterial genomes, using open databases and free bioinformatics tools, can provide an alternative for identification methods used in this research (single-marker gene sequencing and MALDI-TOF protein mass fingerprinting) and deliver more precise and reliable results as well as deeper insight into potential enzymatic abilities of keratinolytic strains.

## 3. Materials and Methods

### 3.1. Sample Collection

All samples were collected during the spring of 2022 from a rural area on the outskirts of Lodz (Lodzkie voivodeship, Central Poland). Six of them consisted of horsehair gathered during the spring shedding season from two warm-blooded mares (*Equus caballus*). Both horses were kept on straw and frequently paddocked throughout the year. For each sample, either winter coat hair, fetlock, or gauze soaked in 0.85% NaCl and swabbed through the entire body was taken from each animal. The six soil samples were collected from a small greenhouse, where fallow deer (*Dama dama*) pelts had been buried approximately 18 months earlier. The soil was dug into 30 cm and 70 cm depths in three places—pelt burial site, and 1 m and 5 m from it (see Table S1). After collection, all samples were stored at 5 °C.

### 3.2. Isolation of Microorganisms

An amount of 0.5 g of each sample or an entire 5 cm × 5 cm gauze was incubated with 10 mL of sterile 5% glucose water for 24 h at 20 °C and 500 rpm. Serial dilutions with sterile 0.85% NaCl (from 10^−1^ to 10^−6^) were prepared for all the samples. Then, 20 μL of each dilution was pipetted onto agar plates with solid Lysogeny Broth (LB) medium (10 g/L tryptone peptone; 10 g/L NaCl; 5 g/L yeast extract; 20 g/L agar) and then 100 U/mL of nystatin (POL-AURA, Morąg, Poland) for the inhibition of fungal growth. The plates were incubated at 10 °C, 20 °C, and 30 °C for 72 h. Every 24 h, the growth of microbial cultures was assessed macroscopically. Single colonies were transferred onto fresh LBP plates using the streaking technique and incubated at temperatures of the original isolation. The process was repeated until pure isolates were obtained. The single microbial colonies were used to prepare glycerol stocks with LB medium (medium/glycerol 1:1 (*v*/*v*)) and frozen at −80 °C until further analysis. Example 1 of an equation:

### 3.3. MALDI-TOF MS—Preliminary Identification of Microorganisms

To discriminate potential pathogenic bacteria, isolates were cultured on LB agar plates for 24 h or 48 h at temperatures corresponding to the isolation conditions. For microbial identification with the MALDI-TOF MS technique and direct smear with the formic acid method, a single colony from each isolate was transferred onto a dedicated steel target plate using a sterile 1 µL inoculation loop. Then, 0.5 μL of 25% formic acid (MERCK, Darmstadt, Germany) was added onto biomass, stirred gently, and left until almost dry. Next, 1 μL of saturated matrix solution (40 mg α-cyano-4-hydroxycinnamic acid (α-CHCA; MERCK, Darmstadt, Germany)) in 1 mL of acetonitrile/ethanol/water 1:1:1 (*v*/*v*) with the final trifluoroacetic acid (TFA; MERCK, Darmstadt, Germany) concentration of 3% was added, mixed in, and left to air-dry for 30 min. The dried samples were then analyzed with an AXIMA-iD Plus Confidence MALDI-TOF mass spectrometer (Kratos Analytical Ltd., Manchester, UK and Shimadzu Corporation, Kyoto, Japan). The spectra were collected using Launchpad 2.9 software (Kratos Analytical Ltd., Manchester, UK and Shimadzu Corporation, Kyoto, Japan) in the linear positive ion mode in the mass-to-charge (m/z) ratio range of 2000 to 20,000 Da, with a 50 Hz laser frequency and 90% of laser power. For each sample, a mass spectrum of 200 profiles, each consisting of 5 laser shots, was acquired. The *E. coli* DH5α (TAKARA BIO Inc., Kusatsu, Japan) cells cultivated overnight on an LB agar plate at 30 °C were used as a control. The SARAMIS Premium 4.11 software (Spectral ARchive and Microbial Identification System; bioMérieux, Craponne, France) was used for comparative analysis of the obtained and reference spectra. Results of the analyses are expressed as confidence scores values [%]. The results obtained in this work are listed in Supplementary Table S1. Only scores ≥ 70% were regarded as sufficient for identification. All potentially pathogenic microorganisms identified among isolates were excluded from further study.

### 3.4. Screening for Proteolytic and Keratinolytic Enzymatic Activities

#### 3.4.1. Activation of Isolates and Inoculum Preparation

Isolated strains were cultivated in 20 mL of standard LB medium, inoculated with 20 μL of previously prepared and thawed glycerol stocks, for 24 h or 48 h with 140 rpm shaking at 20 °C or 30 °C (depending on the isolation temperature; strains isolated at 10 °C were cultivated at 20 °C to shorten incubation time). After incubation, the optical density (OD) of each culture was measured using a UV-VIS spectrophotometer (Shimadzu UV-1800, Kyoto, Japan) at a 660 nm wavelength. The obtained cultures were then centrifuged for 5 min at 5000 rpm. The pellet was washed with sterile distilled water and centrifuged again. The cells were then resuspended in an adequate volume of distilled water to obtain OD_660_ = 1 and used as inoculum for a proteolytic activity assay and keratinolytic activity screening.

#### 3.4.2. Proteolytic Activity Assay

For the semi-quantitative assay of proteolytic activity, 2 μL of inoculum (OD_660_ = 1) was spotted in triplicate on fresh LB agar plates enriched with 0% fat UHT cow’s milk in a 4:1 (*v*/*v*) medium/milk ratio. The plates were incubated at 10 °C, 20 °C, or 30 °C and observed after 24 and 48 h. The clear zones around microbial colonies were measured each time and the enzymatic activity index (EAI) for proteolytic activity was calculated using the provided formula:

$$EAI=\frac{\bar{d_{z}}-\bar{d_{c}}}{\bar{d_{c}}}$$

where

$\bar{d_{z}}$—the average diameter of clear zones around colonies;
$\bar{d_{c}}$—the average diameter of colonies.

For comparative analysis of proteolytic activity among the tested strains, it was assumed that EAI values of ≤0.9, ≤1.8, and ≤2.9 correspond to low, medium, and high enzymatic activities towards casein.

#### 3.4.3. Screening for Keratinolytic Activity

For the qualitative screening of keratinolytic activity, strains were cultivated in 250 mL flasks filled with 50 mL of minimal mineral medium (0.5 g/L NH_4_Cl, 0.5 g/L NaCl, 0.3 g/L K_2_HPO_4_, 0.4 g/L KH_2_PO_4_, 0.1 g/L MgSO_4_, 0.1 g/L yeast extract) with the addition of 1% (*w*/*v*) keratinolytic activity inducer. Each strain was screened with three types of inducers—chicken feathers (CHFs), dog hair (DH), and horsehair (HH). All keratin substrates were degreased beforehand by 30 min of incubation in a chloroform/ethanol 1:1 (*v*/*v*) mixture with intermittent stirring at room temperature and air-dried for 24 h. Keratin inducers were sterilized with liquid media for 15 min at 121 °C. Cultures were inoculated with 1 mL of cells washed and suspended in distilled water with OD_660_ = 1, and incubated on a rotatory shaker (INFORS HT, Bottmingen, Switzerland) for 7 days at 140 rpm at 10 °C, 20 °C, or 30 °C. After incubation, the post-culture liquids were separated from keratin residues using standard vacuum filtration on a Büchnel funnel with 70 mm Munktell filter paper (Chem-Land, Stargard, Poland). The obtained filtrates were stored at −20 °C. Keratin substrate residues were left to air-dry for at least 24 h. To qualitatively assess the keratinolytic abilities of the isolated strains, the changes in the appearance of post-culture liquids and the degree of keratin substrate degradation were observed macroscopically, graded from 0 to 3. It was assumed that a value of 0.5 indicates only a higher turbidity of culture medium; 1 and 1.5 corresponded to a lesser or greater degree of fragmentation of the horsehairs; 2 indicates a significant decomposition and fragmentation of both used substrates; 2.5 corresponds to a nearly complete decomposition of the feathers, where the only remnants are quills; and 3 means complete degradation of the keratin substrate. The tested strains were compared with the negative control samples (without microbial inoculation).

### 3.5. Molecular Identification

To identify or confirm the identification of 29 strains, selected based on their keratinolytic and proteolytic activities, the growth rate and MALDI-TOF MS results were chosen for molecular identification via the amplification and sequencing of the 16S rDNA gene V3-V4 fragment with 27F (5′-AGAGTTTGATCMTGGCTCAG-3′) and 785R (5′-CTACCAGGGTATCTAATCC-3′) primers. Genomic DNA (gDNA) was isolated from overnight single colonies using the GeneMATRIX Bacterial & Yeast Genomic DNA Purification Kit (EURx, Gdańsk, Poland). Polymerase chain reaction (PCR) was performed with OptiTaq DNA polymerase in a ready-to-use tiOptiTaq Master Mix (EURx, Poland) solution. The obtained PCR products were purified with the GeneMATRIX Basic DNA Purification Kit (EURx, Poland) and sequenced with Sanger DNA sequencing at Genomed S.A. (Warsaw, Poland). The sequences were then trimmed and compared with reference sequences from the GenBank database using the basic local alignment search tool (BLAST^®^, NCBI, Bethesda, MD, USA). The sequences obtained in this work were deposited in the GenBank database and their accession numbers are listed in Supplementary Table S2.

### 3.6. Phylogenetic Analysis

Phylogenetic analysis was based on the most accurate fragments of 16S rDNA region v3-v4, which was proven by [75]. A sequence dataset was created by trimming the v3-v4 region from 16S rDNA genes downloaded from GenBank (Supplementary Table S3). Alignments were performed using the MAFFT v.7 program using default parameters [76]. Phylogenetic analyses were performed in the MEGA-X program using the maximum likelihood (ML) method. Bootstraps analyses were performed using ultrafast bootstrap approximation with 1000 replicates.

## 4. Conclusions

Sustainable waste management is an important part of pro-environmental practices. The isolation and characterization of keratinolytic microorganisms are necessary for the development of more feasible and greener keratin waste management technologies, which corresponds perfectly with the findings presented in this article. The authors isolated 113 strains from soil and horsehair samples, which were screened for proteolytic and keratinolytic activity. The keratinolytic strains identified in this study belong mainly to the genera *Bacillus, Pseudomonas,* and *Exiguobacterium* with *Kocuria* and *Stenotrophomonas*. Based on the obtained results, it can be concluded that the soil isolates have higher proteolytic and keratinolytic activities compared to strains from horsehairs. In addition, strains derived from soil can degrade waste substrates rich in α- and β-keratins. There is, however, a marked difference in the degradation of these two substrates, with chicken feathers being more favorable. It is therefore important to look for strains that show activity against different keratin-rich substrates. In further research, a more in-depth genomic, metabolomic, and proteomic characterization of identified keratinolytic strains must be performed for the development of rational and environmentally friendly keratin waste bioprocessing technologies.

## Figures and Tables

**Table 1:** Environmental samples used for the isolation of microorganisms with keratinolytic potential.

Sample Name	Sample Type	Description
KGMa	Winter coat horsehair	Collected during spring shedding from Lesser Poland warm-blooded horse (*Equus caballus*); height 164 cm, bay with slight feathers, born in 1998
KGHa	Winter coat horsehair	Collected during spring shedding from Polish noble half-blood horse (*Equus caballus*), height 172 cm, seal brown; born in 1997
KPMa	Fetlock horsehair	Collected during spring shedding from Lesser Poland warm-blooded horse (*Equus caballus*); height 164 cm, bay with slight feathers, born in 1998
KPH	Fetlock horsehair	Collected during spring shedding from Polish noble half-blood horse (*Equus caballus*), 172 cm, seal brown; born in 1997
KSMa	Full body swab	Full body swab with sterile gauze soaked in 0.9% NaCl from neck, groin, back, withers, and upper rump of Lesser Poland warm-blooded horse (*Equus caballus*); height 164 cm, bay with slight feathers, born in 1998
KSHa	Full body swab	Full body swab with sterile gauze soaked in 0.9% NaCl from neck, groin, back, withers, and upper rump of Polish noble half-blood horse (*Equus caballus*), height 172 cm, seal brown; born in 1997
GA1	Soil	Small greenhouse, burial place of fallow deer (*Dama dama*) pelts, Lodz (Lodzkie voivodeship, Central Poland), depth 30 cm
GA2	Soil	Small greenhouse, burial place of fallow deer (*Dama dama*) pelts, Lodz (Lodzkie voivodeship, Central Poland), depth 70 cm
GB1	Soil	Small greenhouse, burial place of fallow deer (*Dama dama*) pelts, Lodz (Lodzkie voivodeship, Central Poland), depth 30 cm, 5 m away from GA site
GB2	Soil	Small greenhouse, burial place of fallow deer (*Dama dama*) pelts, Lodz (Lodzkie voivodeship, Central Poland), depth 70 cm, 5 m away from GA site
GC1	Soil	Small greenhouse, burial place of fallow deer (*Dama dama*) pelts, Lodz (Lodzkie voivodeship, Central Poland), depth 30 cm, 1 m away from GA site
GC2	Soil	Small greenhouse, burial place of fallow deer (*Dama dama*) pelts, Lodz (Lodzkie voivodeship, Central Poland), depth 70 cm, 1 m away from GA site

## Data Availability

The data are contained within this article.

## Supplementary Materials

The following supporting information can be downloaded at https://www.mdpi.com/article/10.3390/molecules29143380/s1. Table S1: Isolate identification using MALDI-TOF MS and SARAMIS Premium database; Table S2: Taxonomic identification based on v3-v4 16S rDNA fragment analysis with BLASTn^®^ (NCBI); Table S3: The accession numbers of 16S rDNA genes used in the phylogenetic analysis.
