# Supplementary material for: Isolation, Selection, and Identification of Keratinolytic Bacteria for Green Management of Keratin Waste
Source: Molecules. 2024 Jul 18;29(14):3380. doi: 10.3390/molecules29143380 (PMC11280386; doi:10.3390/molecules29143380)
Supplement: Supplementary file 1 [file molecules-29-03380-s001.zip › molecules-3086097-supplementary.pdf]

Isolation, Selection, and Identification of Keratinolytic Bacteria for Green Management of Keratin Waste

Wiktoria Gerlicz [1], Marcin Sypka [1], Iga Jodłowska [1] and Aneta Białkowska [1,*]

[1]Institute of Molecular and Industrial Biotechnology, Faculty of Biotechnology and Food Sciences, Lodz University of Technology, 90-537 Lodz, Poland

Supplementary materials

Supplementary Table S1. Isolates identification using MALDI-TOF MS and SARAMIS Premium database 1
Supplementary Table S2. Taxonomic identification based on v3-v4 16S rDNA fragments analysis with BLASTn® (NCBI) 4
Supplementary Table S3. GenBank accession numbers of the sequences used in this study the phylogenetic analysis. 5

Supplementary Table S1*. Isolates identification using MALDI-TOF MS and SARAMIS Premium database*

Strain	Source of sample	Isolation temp. [°C]	Taxonomic identification							MALDI ID score [%]*
			Domain	Phylum	Class	Order	Family	Genus	Species	
Ker1	KGHa	30	Bacteria	Bacillota	Bacilli	Bacillales	Bacillaceae	*Exiguobacterium*	-	40.4
Ker2	KGMa	30	Unidentified							-
Ker3	KGMa	30	Unidentified							-
Ker4	KGMa	30	Unidentified							-
Ker5	KGMa	30	Unidentified							-
Ker6	KGMa	30	Bacteria	Pseudomonadota	Gammaproteobacteria	Moraxellales	Moraxellaceae	*Acinetobacter*	*lwoffi*	78.7
Ker7	KGMa	30	Unidentified							-
Ker8	KGMa	30	Bacteria	Pseudomonadota	Gammaproteobacteria	Moraxellales	Moraxellaceae	*Acinetobacter*	*lwoffi*	50.6
Ker9a	KGMa	30	Unidentified							-
Ker9b	KGMa	30	Unidentified							-
Ker9c	KGMa	30	Bacteria	Pseudomonadota	Gammaproteobacteria	Moraxellales	Moraxellaceae	*Acinetobacter*	*lwoffi*	78.1
Ker10a	KGMa	30	Unidentified							-
Ker10b	KGMa	30	Unidentified							-
Ker11	KGMa	30	Unidentified							-
Ker12	KGMa	20	Bacteria	Bacillota	Bacilli	Bacillales	Bacillaceae	*Exiguobacterium*		40.9
Ker13	KGMa	20	Bacteria	Bacillota	Bacilli	Bacillales	Bacillaceae	*Bacillus*	*mycoides*	99.9
Ker14	KGHa	20	Unidentified							-
Ker15	KGHa	30	Bacteria	Bacillota	Bacilli	Bacillales	Staphylococcaceae	*Staphylococcus*	*vitulinus*	47.7
Ker16	KGMa	30	Unidentified							-
Ker17	KGMa	30	Bacteria	Bacillota	Bacilli	Bacillales	Bacillaceae	*Exiguobacterium*	-	44.5
Ker18	KGMa	30	Bacteria	Bacillota	Bacilli	Bacillales	Bacillaceae	*Bacillus*	*cereus*	99.9
Ker19	KGMa	30	Bacteria	Bacillota	Bacilli	Lactobacillales	Aerococcaceae	*Aerococcus*	*viridans*	99.9

Ker20	KGMa	30	Bacteria	Bacillota	Bacilli	Lactobacillales	Aerococcaceae	*Aerococcus*	*viridans*	88.0
Ker21	KGHa	30	Bacteria	Bacillota	Bacilli	Bacillales	Staphylococcaceae	*Staphylococcus*	*vitulinus*	49.1
Ker22	KGHa	30	Bacteria	Bacillota	Bacilli	Bacillales	Bacillaceae	*Bacillus*	*subtilis*	78.0
Ker23	KGHa	30	Unidentified							-
Ker24	KGHa	30	Bacteria	Pseudomonadota	Gammaproteobacteria	Enterobacterales	Enterobacteriaceae	*Leclercia*	*adecarboxy lata*	55.3
Ker25	KGMa	10	Bacteria	Bacillota	Bacilli	Bacillales	Bacillaceae	*Exiguobacterium*	-	47.3
Ker26	KGMa	10	Bacteria	Pseudomonadota	Gammaproteobacteria	Moraxellales	Moraxellaceae	*Acinetobacter*	*lwoffi*	88.8
Ker27	KPH	30	Unidentified							-
Ker28	KPH	30	Unidentified							-
Ker29	KGMa	30	Bacteria	Bacillota	Bacilli	Bacillales	Bacillaceae	*Bacillus*	*cereus*	99.9
Ker30	KPH	30	Bacteria	Bacillota	Bacilli	Bacillales	Bacillaceae	*Bacillus*	*mycoides*	87.0
Ker31	KPH	20	Bacteria	Actinomycetota	Actinomycetes	Micrococcales	Micrococcaceae	*Micrococcus*	-	42.5
Ker32	KPMa	20	Bacteria	Bacillota	Bacilli	Bacillales	Bacillaceae	*Bacillus*	*cereus* group	92.0
Ker33	KPH	30	Bacteria	Bacillota	Bacilli	Bacillales	Staphylococcaceae	*Staphylococcus*	*vitulinus*	52.9
Ker34	KPMa	30	Bacteria	Bacillota	Bacilli	Bacillales	Bacillaceae	*Bacillus*	*cereus* group	92.0
Ker35	KPMa	30	Unidentified							-
Ker36	KPMa	30	Bacteria	Bacillota	Bacilli	Bacillales	Bacillaceae	*Exiguobacterium*	-	45.3
Ker37	KPMa	30	Bacteria	Bacillota	Bacilli	Bacillales	Bacillaceae	*Priestia*	*megateriu m*	99.9
Ker38	KPMa	30	Unidentified							
Ker39	KPMa	20	Bacteria	Bacillota	Bacilli	Bacillales	Bacillaceae	*Bacillus*	*cereus* group	96.9
Ker40	KPH	20	Bacteria	Bacillota	Bacilli	Bacillales	Bacillaceae	*Exiguobacterium*	-	43.1
Ker41	KPH	20	Bacteria	Bacillota	Bacilli	Bacillales	Bacillaceae	*Bacillus*	*cereus* group	87.4
Ker42	KPH	30	Bacteria	Actinomycetota	Actinomycetes	Micrococcales	Micrococcaceae	*Arthrobacter*	*arilaitensis*	44.2
Ker43	KPMa	30	Unidentified							-
Ker44	KPH	30	Unidentified							-
Ker45	KPMa	20	Bacteria	Bacillota	Bacilli	Bacillales	Bacillaceae	*Bacillus*	*cereus* group	99.9
Ker46	KPH	20	Bacteria	Actinomycetota	Actinomycetes	Micrococcales	Micrococcaceae	*Arthrobacter*	*bergerei*	61.3
Ker47	KPMa	20	Unidentified							-
Ker48	KPMa	10	Unidentified							-
Ker49	KSHa	30	Bacteria	Bacillota	Bacilli	Bacillales	Bacillaceae	*Bacillus*	*cereus* group	95.0
Ker50	KSMa	20	Bacteria	Bacillota	Bacilli	Bacillales	Bacillaceae	*Bacillus*	*cereus* group	92.0
Ker51	KSHa	30	Bacteria	Bacillota	Bacilli	Bacillales	Bacillaceae	*Bacillus*	*cereus* group	99.9
Ker52	KSHa	30	Bacteria	Bacillota	Bacilli	Lactobacillales	Aerococcaceae	*Aerococcus*	*viridans*	96.4
Ker53	GA1	30	Bacteria	Bacillota	Bacilli	Bacillales	Bacillaceae	*Bacillus*	*cereus* group	99.9
Ker54	GA2	30	Bacteria	Bacillota	Bacilli	Bacillales	Bacillaceae	*Bacillus*	*cereus* group	92.0
Ker55	GA1	30	Bacteria	Bacillota	Bacilli	Bacillales	Bacillaceae	*Bacillus*	*cereus* group	99.9
Ker56	GA2	30	Bacteria	Bacillota	Bacilli	Bacillales	Bacillaceae	*Bacillus*	*cereus* group	99.9
Ker57	GA2	30	Bacteria	Pseudomonadota	Gammaproteobacteria	Aeromonadales	Aeromonadaceae	*Aeromonas*	*media*	77.7

Ker58	GA1	20	Bacteria	Bacillota	Bacilli	Bacillales	Bacillaceae	*Bacillus*	*cereus* group	99.9
Ker59	GA1	20	Bacteria	Bacillota	Bacilli	Bacillales	Bacillaceae	*Bacillus*	*cereus* group	99.9
Ker60	GA2	20	Unidentified							-
Ker61	GA1	30	Bacteria	Bacillota	Bacilli	Bacillales	Bacillaceae	*Bacillus*	*cereus* group	95.0
Ker62	GA1	30	Bacteria	Pseudomonadota	Gammaproteobacteria	Aeromonadales	Aeromonadaceae	*Aeromonas*	-	81.4
Ker63	GA2	30	Bacteria	Bacillota	Bacilli	Bacillales	Bacillaceae	*Bacillus*	*cereus* group	99.9
Ker64	GA1	20	Bacteria	Bacillota	Bacilli	Bacillales	Bacillaceae	*Bacillus*	*cereus* group	99.9
Ker65	GA1	20	Bacteria	Bacillota	Bacilli	Bacillales	Bacillaceae	*Bacillus*	*cereus* group	96.6
Ker66	GA1	20	Bacteria	Pseudomonadota	Gammaproteobacteria	Enterobacterales	Yersiniaceae	*Serratia*	*plymuthica*	74.6
Ker67	GA1	10	Bacteria	Pseudomonadota	Gammaproteobacteria	Pseudomonadales	Pseudomonadaceae	*Pseudomonas*	-	87.6
Ker68	GB1	30	Bacteria	Bacillota	Bacilli	Bacillales	Bacillaceae	*Bacillus*	*cereus* group	86.7
Ker69	GB1	30	Bacteria	Bacillota	Bacilli	Bacillales	Bacillaceae	*Bacillus*	*cereus* group	99.9
Ker70	GB1	30	Bacteria	Bacillota	Bacilli	Bacillales	Bacillaceae	*Bacillus*	*cereus* group	96.9
Ker71	GB1	30	Bacteria	Bacillota	Bacilli	Bacillales	Bacillaceae	*Bacillus*	*mycoides*	79.5
Ker72	GB1	30	Bacteria	Bacillota	Bacilli	Bacillales	Bacillaceae	*Bacillus*	*cereus* group	75.0
Ker73	GB2	30	Bacteria	Bacillota	Bacilli	Bacillales	Bacillaceae	*Bacillus*	*cereus* group	96.9
Ker74	GB2	30	Bacteria	Bacillota	Bacilli	Bacillales	Bacillaceae	*Bacillus*	*cereus* group	96.6
Ker75	GB1	20	Bacteria	Bacillota	Bacilli	Bacillales	Bacillaceae	*Bacillus*	*cereus* group	99.9
Ker76	GB1	20	Bacteria	Bacillota	Bacilli	Bacillales	Bacillaceae	*Bacillus*	*cereus*	44.8
Ker77	GB2	20	Unidentified							
Ker78	GB2	20	Bacteria	Bacillota	Bacilli	Bacillales	Bacillaceae	*Bacillus*	*mycoides*	99.9
Ker79	GB2	30	Unidentified							-
Ker80	GB2	30	Unidentified							-
Ker81	GB2	30	Unidentified							-
Ker82	GB1	30	Bacteria	Bacillota	Bacilli	Bacillales	Bacillaceae	*Bacillus*	*cereus* group	96.6
Ker83	GB1	30	Unidentified							-
Ker84	GB1	30	Bacteria	Bacillota	Bacilli	Bacillales	Bacillaceae	*Bacillus*	*cereus* group	96.9
Ker85	GB1	30	Bacteria	Bacillota	Bacilli	Bacillales	Bacillaceae	*Bacillus*		56.5
Ker86	GB2	20	Bacteria	Pseudomonadota	Gammaproteobacteria	Aeromonadales	Aeromonadaceae	*Aeromonas*	-	99.9
Ker87	GB1	20	Bacteria	Pseudomonadota	Gammaproteobacteria	Pseudomonadales	Pseudomonadaceae	*Pseudomonas*	*lini*	60.3
Ker88	GB1	20	Unidentified							-
Ker89	GB1	30	Unidentified							-
Ker90	GB2	30	Unidentified							-
Ker91	GB1	20	Bacteria	Bacillota	Bacilli	Bacillales	Bacillaceae	*Bacillus*	*cereus* group	87.4
Ker92	GC2	30	Unidentified							-
Ker93	GC2	30	Bacteria	Pseudomonadota	Gammaproteobacteria	Aeromonadales	Aeromonadaceae	*Aeromonas*	-	78.4
Ker94	GC2	10	Bacteria	Pseudomonadota	Gammaproteobacteria	Aeromonadales	Aeromonadaceae	*Aeromonas*	-	95.9

Ker95	GC2	30	Bacteria	Bacillota	Bacilli	Bacillales	Staphylococcaceae	*Staphylococcus*	*epidermidis*	95
Ker96	GC1	30	Unidentified							-
Ker97	GC2	20	Bacteria	Bacillota	Bacilli	Bacillales	Bacillaceae	*Bacillus*	*cereus* group	96.9
Ker98	GC1	20	Unidentified							-
Ker99	GC1	20	Bacteria	Bacillota	Bacilli	Bacillales	Bacillaceae	*Bacillus*	*mycoides*	56.7
Ker100	GC1	30	Bacteria	Bacillota	Bacilli	Bacillales	Bacillaceae	*Bacillus*	*cereus* group	96.6
Ker101	GC1	30	Bacteria	Bacillota	Bacilli	Bacillales	Bacillaceae	*Priestia*	*megaterium*	99.9
Ker102	GC2	30	Bacteria	Bacillota	Bacilli	Bacillales	Bacillaceae	*Bacillus*	*cereus* group	87.4
Ker103	GC2	30	Bacteria	Actinomycetota	Actinomycetes	Micrococcales	Micrococcaceae	*Kocuria*	-	77.7
Ker104	GC2	10	Unidentified							-
Ker105	GC1	10	Bacteria	Bacillota	Bacilli	Bacillales	Paenibacillaceae	*Paenibacillus*	*durus*	55.5
Ker106	GC1	20	Unidentified							-
Ker107a	GC1	10	Bacteria	Pseudomonadota	Gammaproteobacteria	Pseudomonadales	Pseudomonadaceae	*Pseudomonas*	-	98.1
Ker107b	GC1	30	Bacteria	Pseudomonadota	Gammaproteobacteria	Xanthomonadales	Xanthomonadaceae	*Stenotrophomonas*	*rhizophila*	66.7
Ker108	GC1	30	Bacteria	Pseudomonadota	Gammaproteobacteria	Pseudomonadales	Pseudomonadaceae	*Pseudomonas*	*tolaasii*	53.6
Ker109	GC1	30	Bacteria	Bacillota	Bacilli	Bacillales	Bacillaceae	*Bacillus*	-	77.1

*** only scores ≥70% are regarded as sufficient for identification;**

Supplementary Table S2. *Taxonomic identification based on v3-v4 16S rDNA fragments analysis with BLASTn® (NCBI)*

**Strain**	**GenBank accession number**	**Taxonomic identification**	
		**Phylum**	**Genus**
Ker2	OR826701.1	Bacillota	*Exiguobacterium*
Ker7	OR826702.1	Bacillota	*Exiguobacterium*
Ker10a	OR826703.1	Bacillota	*Exiguobacterium*
Ker11	OR826704.1	Bacillota	*Exiguobacterium*
Ker31	OR826705.1	Bacillota	*Exiguobacterium*
Ker33	OR826706.1	Bacillota	*Mammaliicoccus*
Ker37	OR826707.1	Bacillota	*Priestia*
Ker43	OR826708.1	Actinomycetota	*Curtobacterium*
Ker62	OR826709.1	Pseudomonadota	*Aeromonas*
Ker67	OR826710.1	Pseudomonadota	*Pseudomonas*
Ker77	PP346379.1	Bacillota	*Bacillus*
Ker79	OR826711.1	Bacillota	*Priestia*
Ker80	OR826712.1	Bacillota	*Bacillus*
Ker81	OR826713.1	Bacillota	*Bacillus*
Ker83	PP346380.1	Bacillota	*Bacillus*
Ker84	OR826714.1	Bacillota	*Bacillus*
Ker85	OR826715.1	Bacillota	*Bacillus*
Ker87	OR826716.1	Pseudomonadota	*Pseudomonas*
Ker88	OR826717.1	Bacillota	*Bacillus*

Ker90	PP346381.1	Bacillota	*Bacillus*
Ker92	OR826718.1	Bacillota	*Bacillus*
Ker94	OR826719.1	Pseudomonadota	*Aeromonas*
Ker96	OR826720.1	Bacillota	*Bacillus*
Ker101	OR826721.1	Bacillota	*Priestia*
Ker103	OR826722.1	Actinomycetota	*Kocuria*
Ker104	OR826723.1	Pseudomonadota	*Pseudomonas*
Ker107a	PP346382.1	Pseudomonadota	*Pseudomonas*
Ker107b	PP346383.1	Pseudomonadota	*Stenotrophomonas*
Ker108	PP346384.1	Pseudomonadota	*Pseudomonas*

Supplementary Table S3. *GenBank accession numbers of the sequences used in this study the phylogenetic analysis.*

Species	Strain number	GenBank accession number
Exiguobacterium indicum	HHS 31	NR_042347.1
Exiguobacterium acetylicum	NBRC 12146	NR_113585.1
Exiguobacterium artemiae	9AN	NR_114970.2
Exiguobacterium sibiricum	255-15	NR_075006.1
Exiguobacterium antarcticum	DSM 14480	NR_043476.1
Exiguobacterium undae	L2	NR_114811.1
Mammaliicoccus vitulinus	ATCC 51145	NR_024670.1
Mammaliicoccus lentus	MAFF 911385	NR_043418.1
Mammaliicoccus sciuri	DSM 20345	NR_025520.1
Mammaliicoccus stepanovicii	196	NR_117252.1
Priestia aryabhattai	B8W22	NR_118442.1
Priestia flexa	IFO15715	NR_024691.1
Priestia megaterium	ATCC 14581	NR_117473.1
Priestia veravalensis	SGD-V-76	NR_178610.1
Priestia qingshengii	G19	NR_133978.1
Curtobacterium allii	20TX0166	NR_181874.1
Curtobacterium flaccumfaciens	BCCM/LMG 3645	NR_025467.1
Curtobacterium luteum	DSM 20542	X77437
Curtobacterium citreum	DSM 20528	X77436
Curtobacterium pusillum	DSM 20527	NR_042315.1
Aeromonas rivipollensis	P2G1	NR_144574.1
Aeromonas veronii bv. veronii	ATCC 35624	NR_119045.1
Aeromonas lacus	AE122	NR_136831.1
Aeromonas australiensis	266	NR_108872.1
Aeromonas hydrophila	K12	GU826155.1
Pseudomonas wadenswilerensis	ID2	NR_157778.1
Pseudomonas donghuensis	HYS	NR_136501.2
Pseudomonas libanensis	CIP 105460	NR_024901.1
Pseudomonas germanica	FIT28	NR_181838.1

Pseudomonas huaxiensis	WCHPs060044	NR_180103.1
Pseudomonas stutzeri	K4	HM756166.1
Bacillus wiedmannii	FSL W8-0169	NR_152692.1
Bacillus tropicus	MCCC 1A01406	NR_157736.1
Bacillus pacificus	MCCC 1A06182	NR_157733.1
Bacillus proteolyticus	MCCC 1A00365	NR_157735.1
Bacillus luti	MCCC 1A00359	NR_157730.1
Bacillus nitratireducens	MCCC 1A00732	NR_157732.1
Bacillus cereus	ATCC 14579	MG708176.1
Bacillus subtilis	IAM 12118	NR_112116.2
Bacillus amyloliquefaciens	NBRC 15535	NR_041455.1
Bacillus licheniformis	ATCC 14580	NR_074923.1
Kocuria tytonis	442	NR_171491.1
Kocuria arsenatis	CM1E1	NR_148610.1
Kocuria rhizophila	TA68	NR_026452.1
Kocuria tytonicola	489	NR_179862.1
Stenotrophomonas rhizophila	e-p10	NR_121739.1
Stenotrophomonas nematodicola	W5	NR_181111.1
Stenotrophomonas bentonitica	BII-R7	NR_157765.1
Stenotrophomonas tumulicola	T5916-2-1b	NR_148818.1
Stenotrophomonas maltophila	BBE11-1	JQ619623.1
